# *Pseudomonas fluorescens* ATCC 13525 Containing an Artificial Oxalate Operon and *Vitreoscilla* Hemoglobin Secretes Oxalic Acid and Solubilizes Rock Phosphate in Acidic Alfisols

**DOI:** 10.1371/journal.pone.0092400

**Published:** 2014-04-04

**Authors:** Kavita Yadav, Chanchal Kumar, G. Archana, G. Naresh Kumar

**Affiliations:** 1 Molecular Microbial Biochemistry Laboratory, Department of Biochemistry, Faculty of Science, The Maharaja Sayajirao University of Baroda, Vadodara, India; 2 Department of Microbiology and Biotechnology Centre, Faculty of Science, The Maharaja Sayajirao University of Baroda, Vadodara, India; Loyola University Medical Center, United States of America

## Abstract

Oxalate secretion was achieved in *Pseudomonas fluorescens* ATCC 13525 by incorporation of genes encoding *Aspergillus niger* oxaloacetate acetyl hydrolase (*oah*), *Fomitopsis plaustris* oxalate transporter (*FpOAR*) and *Vitreoscilla* hemoglobin (*vgb*) in various combinations. *Pf* (pKCN2) transformant containing *oah* alone accumulated 19 mM oxalic acid intracellularly but secreted 1.2 mM. However, in the presence of an artificial oxalate operon containing *oah* and *FpOAR* genes in plasmid pKCN4, *Pf* (pKCN4) secreted 13.6 mM oxalate in the medium while 3.6 mM remained inside. This transformant solubilized 509 μM of phosphorus from rock phosphate in alfisol which is 4.5 fold higher than the *Pf* (pKCN2) transformant. Genomic integrants of *P. fluorescens* (*Pf* int1 and *Pf* int2) containing artificial oxalate operon (*plac-FpOAR-oah*) and artificial oxalate gene cluster (*plac-FpOAR-oah, vgb, egfp*) secreted 4.8 mM and 5.4 mM oxalic acid, released 329 μM and 351 μM P, respectively, in alfisol. The integrants showed enhanced root colonization, improved growth and increased P content of *Vigna radiata* plants. This study demonstrates oxalic acid secretion in *P. fluorescens* by incorporation of an artificial operon constituted of genes for oxalate synthesis and transport, which imparts mineral phosphate solubilizing ability to the organism leading to enhanced growth and P content of *V. radiata* in alfisol soil.

## Introduction

Acidic soils occupy about 30% of world's ice-free land area and are considered important for future agricultural development, as presently only 24.2% of the total land area of the world is potentially arable [1], [2]. Phosphorous (P) is the second major nutrient in the soil limiting plant growth after nitrogen (N). In acidic alfisols, plant growth and crop yields are limited by low P availability combined with high refixation of applied P [3]–[5]. Due to the high reactivity of soluble inorganic phosphate (Pi) with Al, Fe and Ca, most soil P exists in the bound form and very low (< 10 μM) amount of free P is available for plant growth in soil solution [6]. In acidic alfisols, P is mainly complexed with Fe and Al [7], [8] which are difficult to dissolve by simple acidification. However, organic acids have a demonstrated chelation capacity, making them potentially ideal for releasing P from alfisols [5].

Application of rock phosphate (RP) as a P fertilizer to acidic soils is considered as an important strategy for enhancing plant P nutrition [9]. RP is rich in mineral phosphate complexes which could be solubilized in acidic conditions in alfisols. However, partially acidulated rock phosphate (PARP) obtained by mild acid treatment of RP, renders it more easily available for plants [10], indicating that mere addition of untreated RP to alfisols is not efficient. An alternative approach is to use organic acid secreting microorganisms to solubilize RP in alfisols [11], [12]. Low molecular weight organic acids are known to be most effective in chelation of Fe and Al and thus solubilization of P [13]–[15]. Plants secreting piscidic, citric and oxalic acid in root exudates show increased growth and shoot P content [15]–[17]. Improved growth of pigeon pea plants as compared to other crops in alfisols is attributed to piscidic acid mediated FeP solubilization [7].

Although many microorganisms are known to solubilize mineral phosphates [6], majority of them solubilize Ca-P and very few microorganisms are known to solubilize Fe-P and Al-P [18]. As a result most phosphate solubilizing microorganisms are ineffective in supplying P to plants grown in alfisols. For instance, *Enterobacter asburiae* PSI3, a gluconic acid secreting bacterium, solubilizes P from alkaline vertisols [19] but does not release free P from alfisols supplemented with RP [5]. This has been attributed to the nature and amount of the organic acid secreted by the microorganism. It has been shown that P is released efficiently from alfisol amended with RP when treated with organic acids such as oxalic and citric as compared to gluconate, succinate and malate which are more effective in vertisol or alkaline soils [5]. About 5–10 mM oxalic acid solubilizes P from RP in acidic alfisol which could be accredited to the excellent chelating properties of this acid that plausibly hinders refixation of P by chelation of Fe and Al ions [5], [15], [20]. Addition of oxalate to different phosphate rocks and soils resulted in efficient mineral phosphate solubilization [5], [15].

Only a few bacterial strains belonging to *Bacillus subtilis*, *Pseudomonas fluorescens*, *Arthrobacter* spp. and *Micrococcus* spp. are known to secrete oxalic acid that too in very low amounts (∼ 2 mM) [21], [22]. High levels of oxalic acid secretion are reported in fungi such as *Aspergillus niger*, *A. fumigatus*, *Botrytis cinerea*, *Fomitopsis plaustris* and *Penicillium* spp. [21]. In fungi, oxalic acid synthesis is mediated by the cytosolic enzyme, oxaloacetate acetyl hydrolase (OAH), which breaks down oxaloacetate into oxalic and acetic acids [23]–[26]. *A. niger* OAH is a pH inducible enzyme belonging to PEP mutase/isocitrate lyase super family and requires divalent metal ions for catalysis [23], [25]–[27]. In bacteria such as *Oxalobacter formigens* oxalate specific transporter (OxlT) is responsible for oxalate uptake and helps in ATP generation [28], [29]. On the other hand, a high amount of oxalate secretion in fungi is mediated by efficient oxalate transporter [30]. *F. plaustris* is a wood rotting fungus and degradation of wood is promoted by oxalic acid secretion with the help of an oxalate transporter encoded by *FpOAR* gene.

Oxygen is present in limited amounts in the rhizosphere which could limit the colonization and survival of rhizobacteria [31]. The obligate aerobic bacterium, *Vitreoscilla*, synthesizes elevated quantities of homodimeric hemoglobin (VHb) under hypoxic growth conditions which allows improved growth under microaerobic conditions when dissolved oxygen is less than 2% of air saturation [32], [33]. Expression of *vgb* gene encoding VHb protein in heterologous hosts often enhances growth and metabolism by facilitating oxygen transfer to the respiratory membranes [34]. Beneficial effect of *vgb* overexpression for improved bacterial growth has been demonstrated in plant associated bacteria [31].

Fluorescent pseudomonads are well-known plant-growth promoting rhizobacteria with root colonization and efficient biocontrol properties [35], [36]. The present study deals with the genetic modification of *Pseudomonas fluorescens* ATCC 13525 for oxalic acid secretion by the incorporation of *A. niger oah* and *F. plaustris FpOAR* genes and determination of its effect on its mineral phosphate solubilizing (MPS) ability and growth promotion of mung bean (*Vigna radiata*) plants in acidic alfisols. Additionally, *vgb* gene was incorporated as a part of an artificial operon containing the *oah* and *FpOAR* genes to enhance the survival and colonization of organism in the soil environment. Incorporation of the artificial oxalate operon in *P. fluorescens* ATCC 13525 resulted in secretion of high amounts of oxalate which in turn released P from RP in acidic alfisols. Incorporation of *vgb* gene along with artificial oxalate operon resulted in better colonization and improved plant parameters in acidic alfisols.

## Materials and Methods

### Bacterial strains, plasmids and media

The plasmids, bacterial and fungal strains used in this study are shown in **Table S1**. Routine DNA manipulations were done with *E. coli* DH10B (Invitrogen, Carlsband, CA, USA) as a host using standard molecular biology protocols [37]. pUC18T-mini-Tn7T-Gm-*eyfp* was generously gifted by Dr. H. P. Schweizer, Colorado State University, USA (**Table S1**) [38]. *P. fluorescens* ATCC 13525 (*Pf*13525) and its plasmid derivatives were grown at 30°C and maintained on *Pseudomonas* agar (Hi Media, India) containing 50 μg/ml ampicillin and 10 μg/ml gentamycin as and when required. Fungal cultures were grown in minimal medium at 27°C [25], [30].

### Construction of artificial oxalate operon

RNA was isolated from *A. niger* by TRizol method (Sigma Aldrich, India) and *oah* gene was amplified from mRNA using gene specific primers (Integrated DNA Technology, USA) (**Table S2**). Amplicon was digested with *Bam*HI/*Pst*I and cloned in *Bam*HI/*Pst*I digested plasmid pUCPM18Gm under *lac* promoter (**Figure S1**). The resultant construct was designated as pKCN2. The RNA isolated from *F. plaustris* was amplified using gene specific primers of *FpOAR* gene (**Table S2**). Amplicon digested with *Sac*I/*Bam*HI was cloned upstream of *oah* gene, in *Sac*I/*Bam*HI digested plasmid pKCN2 under *lac* promoter to construct artificial oxalate operon, designated as pKCN4 (**Figure S1**). Amplicon of 2.8 kb containing *lac-FpOAR-oah* was amplified with forward *lac* primer (**Table S2**) and *oah* reverse primer from pKCN4, using XT-20 polymerase (Merck Genei, India) and was cloned in *Sma*I digested integration vector pUC18T-mini-Tn7T-Gm-*eyfp*.

### Construction of artificial oxalate gene cluster (*plac*-*FpOAR-oah*, *vgb*, *egfp*)

pUCVHb-*egfp* plasmid was digested with *Pvu*II to obtain 3.2 kb insert containing *vgb* and *egfp* genes. Insert was cloned in pKCN5 digested with *Nhe*I and end filled using *Klenow* fragment (Thermo Scientific, USA). The resultant construct (artificial oxalate gene cluster) containing *F. plaustris FpOAR* and *A. niger oah* genes under *lac* promoter, *vgb* gene under its natural oxygen sensitive promoter and *egfp* under *rrnB* promoter in pUC18T-mini-Tn7T-Gm-*eyfp* vector, was designated as pKCN7 (**Figure S1**). All the plasmids were transformed in *Pf*13525 using modified NaCl/CaCl_2_ method [39] and integration in the genome of *Pf*13525 was done by transformation method [40].

### Physiological and analytical experiments

Bacterial inoculum was used to inoculate Tris rock phosphate (TRP) minimal medium [41] and alfisol soil medium (containing 0.5 g/ml alfisol in sterile medium containing 100 mM glucose, 10 mM KNO_3_, micronutrient cocktail and 30 mg RP/g of soil) for batch studies in 150 ml conical flask containing 30 ml of inoculated medium. The culture supernatants collected at the end were used for extracellular organic acid analysis by Prominence UFLC (Shimadzu, Japan) and P estimation [42]. Cell free extract was used for intracellular organic acid analysis.

### MPS ability of *Pf*13525 transformants and integrants

MPS ability of *Pf*13525 transformants and integrants was determined on TRP minimal medium plates (containing 100 mM Tris buffer pH-8.0, 1% methyl red, 1.8% agar and 50 mM glucose) with RP as the sole P source (1mg/ml), respectively. Saline washed bacterial inoculum (5 μl) was spot inoculated on plates and incubated at 30°C. Phosphate solubilization and acid secretion was determined by monitoring the growth and red zone of acidification.

### OAH assay

Cells grown in M9 minimal medium were used for cell free extract preparation and OAH enzyme activity measurements were done by the method described by Lenz et al (1976) [23]. OAH enzyme specific activity was expressed in nmole per minute per mg total protein. Total protein was estimated using a modified Lowry's method [43]. One unit of enzyme activity was defined as the amount of protein required to convert 1 nmole of substrate per minute.

### Plant experiments

Plant studies were done in Murashige and Skoog medium as well as in alfisol soil (containing 10 mg/g of RP). Soil analysis was done from Pulse Research Station (Anand Agriculture University, Vadodara) and was found to contain 0.085% organic carbon, 165.1 kg/ha total nitrogen, 262.4 kg/ha available K and 17.9 kg/ha available P. *V. radiata* (mung bean) plant studies were done as described in [44] and plant parameters such as lengths of the main root and shoot, dry weight and P content were monitored. Molybdate-blue method [45] was used to determine P content. Root colonization of *Pf*13525 integrant was observed on 5^th^ and 10^th^ day after inoculation and different sections of root were observed for bacterial colonization by using Confocal laser scanning microscopy (LSM 700 Carl Zeiss, GmbH).

### Data analysis

Physiological experiments were done in three to four independent replicates for batch culture study. Data are expressed in mean with standard deviation. In plant experiments, three independent triplicate studies were carried out. Differences in mean values were determined using general analysis of variance (ANOVA) and linear regression analysis. The statistical analysis of all the parameters has been done using Graph Pad Prism (version 5.0) software.

## Results

### Effect of genetic modifications on OAH activity, growth and MPS phenotype of *Pf*13525

**Figure 1:**
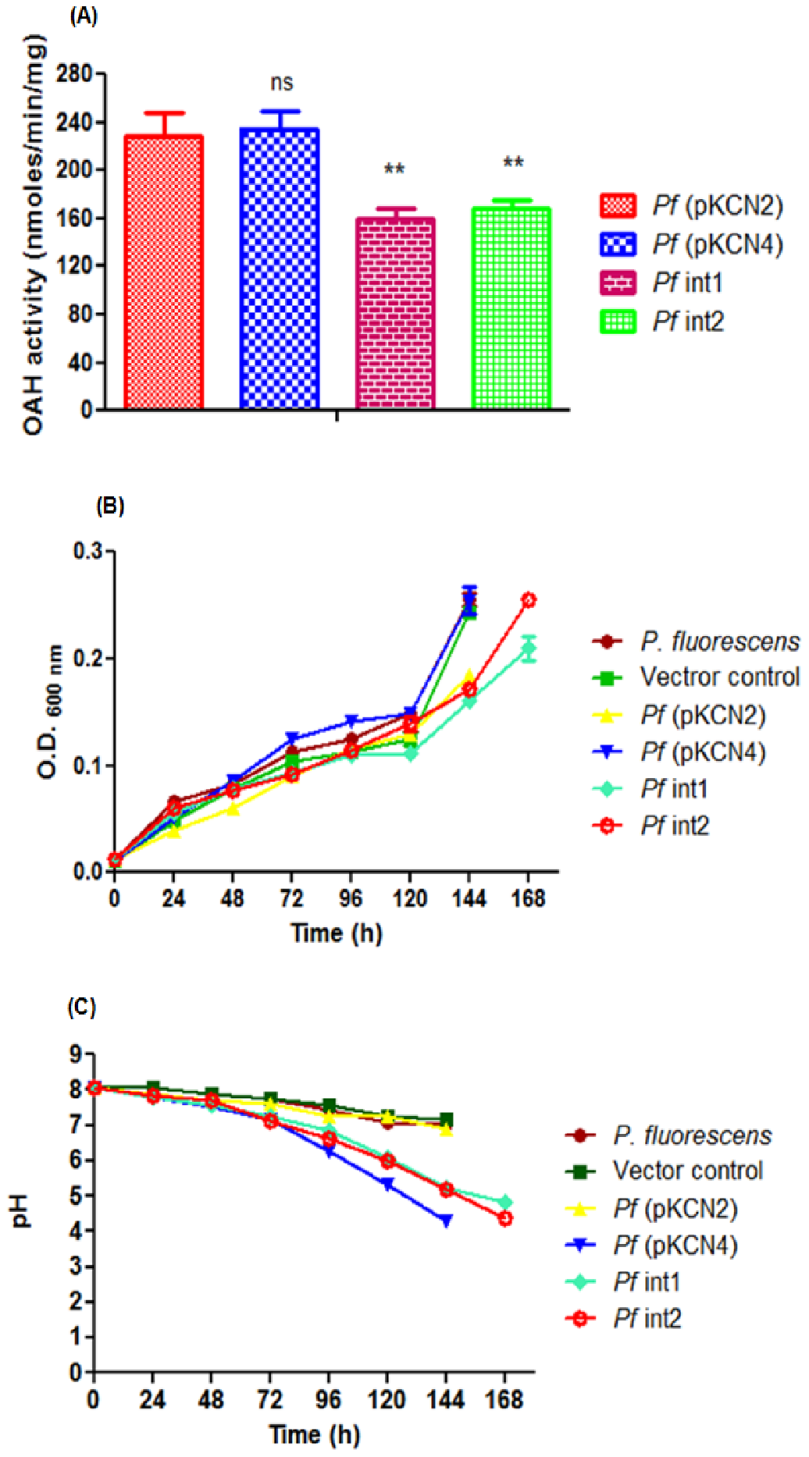
OAH activity, growth and pH profiles of *Pf*13525 wild type and genetically modified strains. **(A)** OAH activity in M9 minimal medium. Results are given as mean ± SD of three independent observations. Values are compared with *Pf*(pKCN2), **, P < 0.0067; ns, non-significant; **(B)** Growth profile and **(C)** pH profile in TRP minimal medium containing 100 mM glucose as carbon source. O.D._600nm_ and pH values at each time point are represented as the mean ± SD of four to six independent observations.

**Figure 2:**
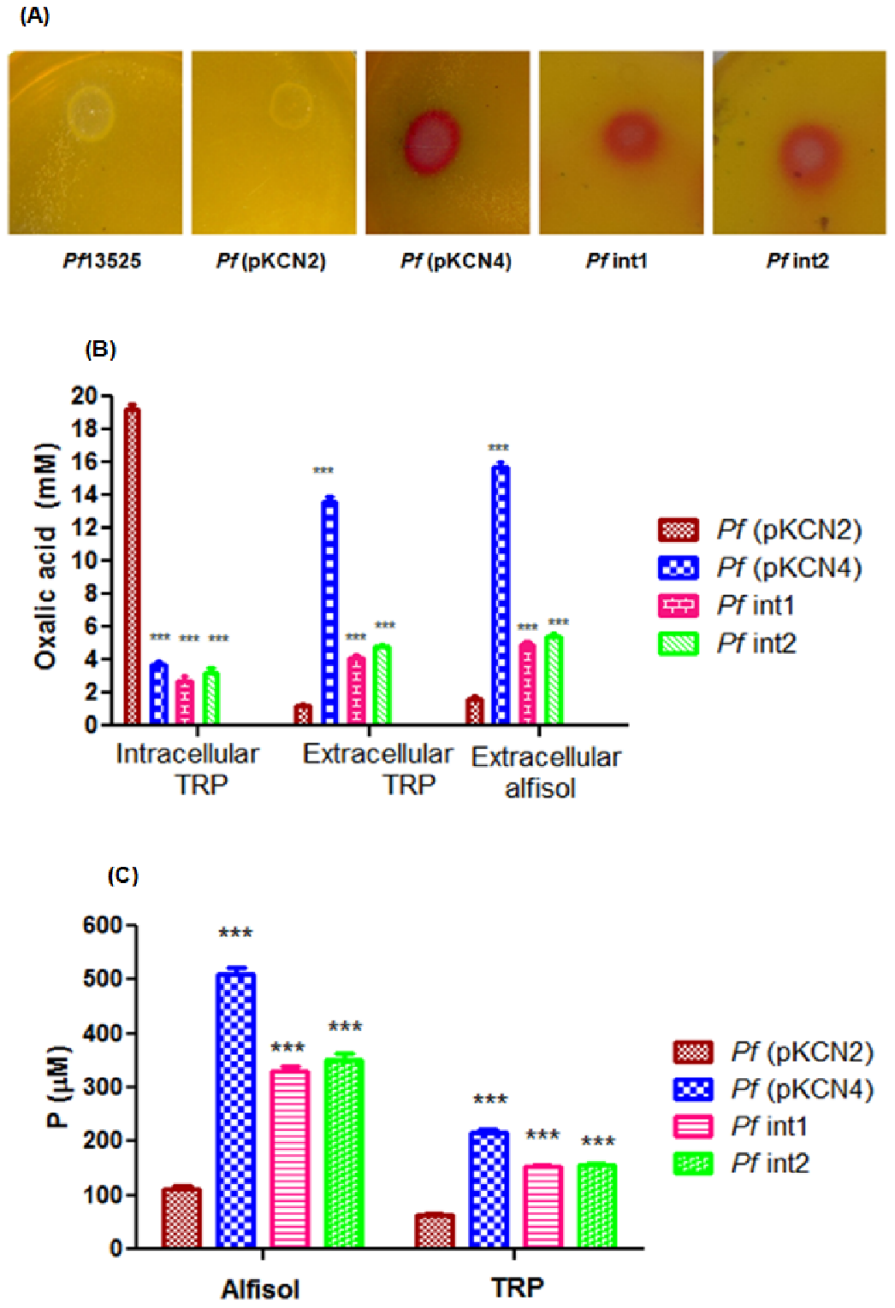
Effect of genetic manipulation on MPS phenotype, oxalic acid secretion and P release by *Pf*13525 strains. **(A)** Phenotype of *Pf*13525 strains on TRP-methyl red agar plates containing 50 mM Tris-HCl and 100 mM glucose. Red zone indicates acidification of the medium; **(B)** Oxalic acid secretion and **(C)** P released by *Pf*13525 integrants and transformants in TRP medium (with 50 mM Tris HCl) and alfisol soil containing 100 mM glucose as carbon source. Results are given as mean ± SD of four to six independent observations where ***, P< 0.0001; ns, non-significant.

*Pf* (pKCN2) and *Pf* (pKCN4) (**Table S1**) transformants showed around 230 U/mg of OAH activity while integrants *Pf* int1 and *Pf* int2 showed about 165 U/mg of OAH activity in M9 minimal medium containing 100 mM glucose (**Figure 1A**). On the other hand, *Pf*13525 and the vector control did not show detectable OAH activity as the gene is absent in the organism. Growth of *Pf* (pKCN4) transformant was not significantly different than the untransformed strain in 50 mM Tris-HCl (pH 8.0) medium containing 100 mM glucose as carbon source and RP as the sole P source (**Figure 1B**). However, the *Pf* (pKCN2) transformant and the integrants grew slowly and reached 0.28 O.D_600 nm_ after 168 h. *Pf* (pKCN4) transformant was most effective in acidification of the medium from pH 8.0 to 4.2 while *Pf* (pKCN2) did not decrease the pH to less than 7.0 (**Figure 1C**). Although growth of the integrants was slower as compared to the pKCN4 transformant, they were effective at reducing the pH of the medium indicating organic acid secretion. Further, organic acid mediated acidification by genetically modified *Pf*13525 strains was observed on TRP plates. *Pf*13525 and *Pf* (pKCN2) transformant did not show red zone of acidification while *Pf* (pKCN4) transformant, *Pf* int1 and *Pf* int2 acidified TRP agar plate containing 100 mM glucose as carbon source and 50 mM Tris HCl (pH 8.0) (**Figure 2A**).

### Effect of genetic modifications on organic acid secretion by *Pf*13525

To study the effect of increased OAH activity on organic acid secretion in genetically modified *Pf*13525 strains, the cell lysate and extracellular culture supernatants were analyzed for oxalic acid levels using HPLC. *Pf* (pKCN2) transformants carrying the *oah* gene showed the highest intracellular accumulation of oxalic acid up to 19.1 mM, but secreted a relatively less amount (1.2 mM) in the medium (**Figure 2B**). On the other hand, *Pf* (pKCN4) transformants possessing artificial oxalate operon, consisting of *oah* gene along with the fungal oxalate transporter, accumulated only 3.6 mM oxalic acid and secreted 13.6 mM in the medium. Genomic integrants of *Pf*13525, int1 and int2 secreted 4.1 and 4.7 mM of oxalic acid, respectively, while intracellular levels were 2.6 and 3.1 mM, respectively (**Figure 2B**).

In order to study the organic acid secretion in soil conditions, alfisol soil supplemented with 100 mM glucose and 30 mg RP/g of soil was inoculated with genetically modified *Pf*13525 strains and the oxalate secreted in the soil solution was estimated. As seen in **Figure 2B**, in agreement with the TRP medium studies, in alfisol *Pf* (pKCN4) secreted 15 mM of oxalate. Oxalate secretion by integrants in alfisol was also comparable to that on TRP medium. Wild type *Pf*13525 secreted 2.0 mM gluconic acid under similar growth conditions but did not show either oxalate accumulation or secretion. All genetic modifications resulted in a decrease in gluconic acid secretion.

### Effect of genetic modifications of *Pf*13525 on MPS ability

In TRP minimal medium, *Pf* (pKCN2), *Pf* (pKCN4), *Pf* int1 and *Pf* int2 released 62 μM, 217 μM, 152 μM and 155 μM of P from RP, respectively (**Figure 2C**). In alfisol soil medium containing 100 mM glucose as the carbon source and supplemented with 30 mg RP/g of soil, *Pf* (pKCN4), *Pf* int1 and *Pf* int2 released 509 μM, 329 μM and 352 μM levels of P, respectively (**Figure 2C**).

### Root colonization study

**Figure 3:**
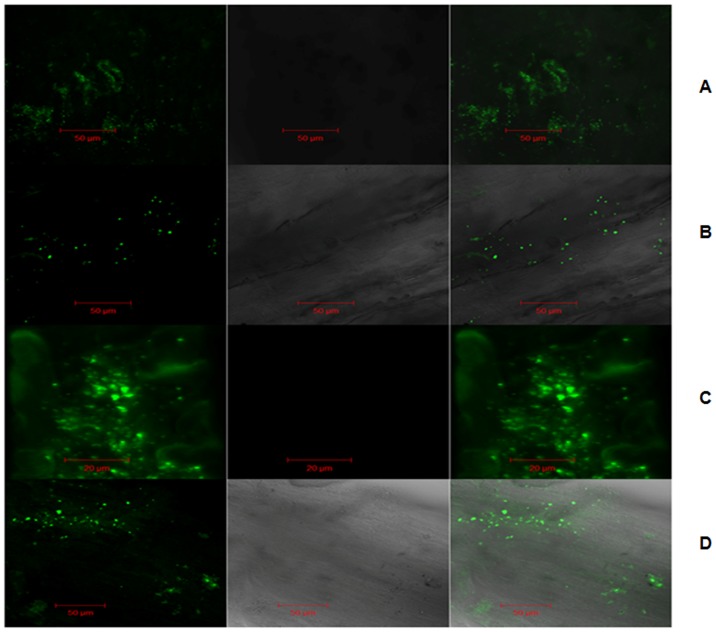
Root colonization study of *V. radiata* inoculated with *Pf* int2. **(A)** and **(B)** colonization study in Murashige-Skoog (MS) medium (hydroponics study) on 5^th^ and 10^th^ day respectively; **(C)** and **(D)** in alfisol soil (pot experiment) on 5^th^ and 10^th^ day, respectively, by Confocal Laser Scanning Microscopy (CLSM). Left panel shows fluorescence imaging, middle panel shows bright field images and rightmost panel shows overlapped images.

Root colonization ability of *Pf* int2 was observed in *V. radiata* plants in Murashige-Skoog's medium and alfisol soil. On 5^th^ day, in both media abundant colonization was seen on the root surfaces, root tips and at branching points and the colonization decreased on 10^th^ day (**Figure 3**).

### Effect of inoculation of genetically modified *Pf*13525 strains on growth parameters of *V. radiata*

**Figure 4:**
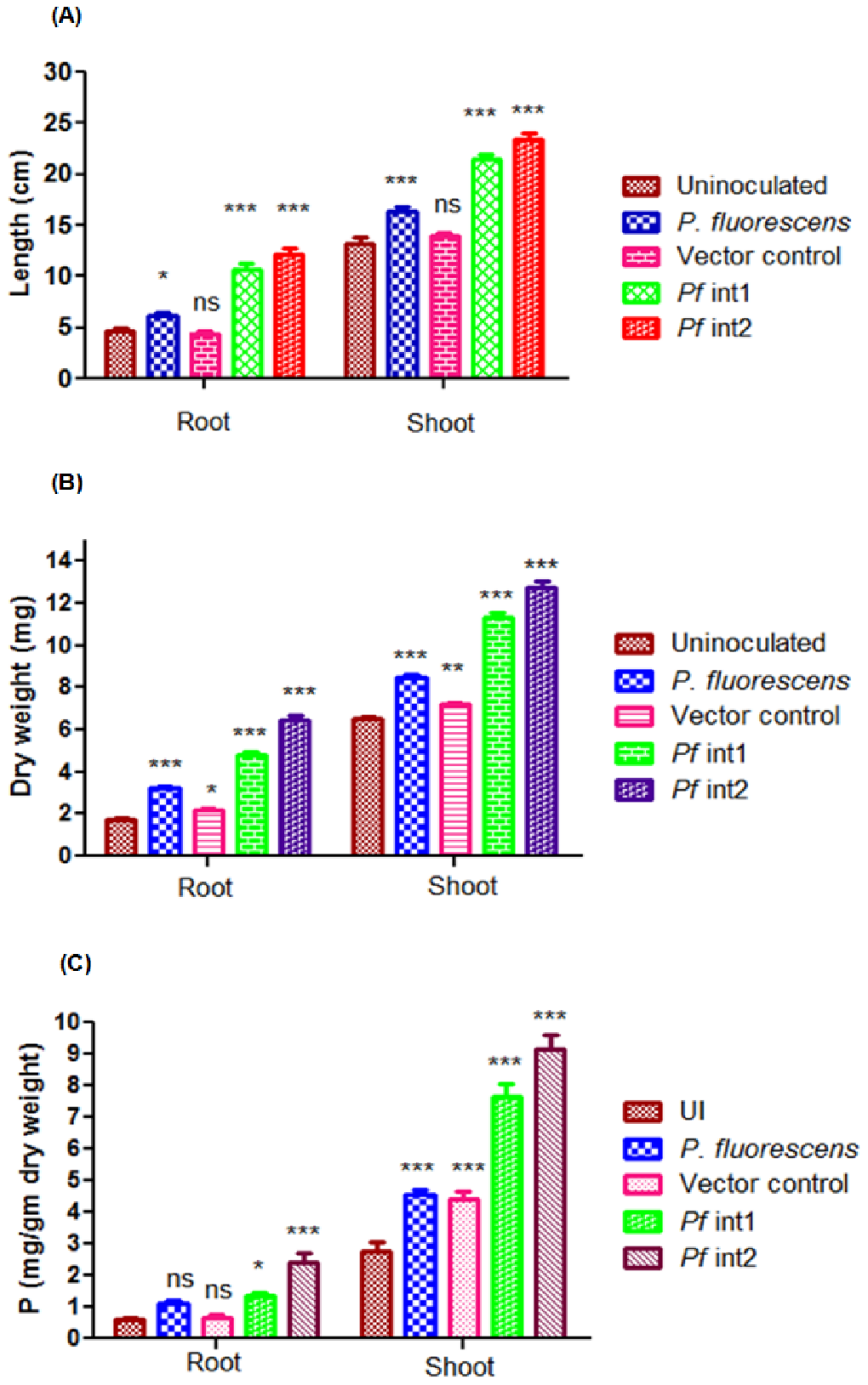
Plant parameters and P content of *V. radiata* plants inoculated with *Pf*13525 wild type and genetically modified strains. **(A)** Plant length; **(B)** Dry weight and **(C)** P content of inoculated *V. radiata* plants in alfisol soil. Values are represented as Mean ± SD of n = 9 observations for plant length and weight while n = 4 to 8 for P content analysis. *, P < 0.05; **, P<0.01; ***, P<0.001; ns, non-significant as compared to the uninoculated control.

Inoculation of *V. radiata* with *Pf* int1 and *Pf* int2 in pot experiments with unsterilized alfisol soil supplemented with RP showed better growth (root and shoot length) and increased root and shoot dry weight. *Pf* int1 and *Pf* int2 inoculations showed 1.7 and 1.9 fold increase in root length, 1.3 and 1.4 fold increase in shoot length of *V. radiata*, respectively, as compared to wild type inoculations (**Figure 4A**). *Pf* int1 and *Pf* int2 inoculations resulted in 1.5 and 2 fold increase in root dry weight and 1.3 and 1.4 fold increase in shoot dry weight, respectively (**Figure 4B**). P content in *Pf* int1 and *Pf* int2 inoculations increased in shoot and root by 1.8 and 2.1 fold, respectively, as compared to plants inoculated with wild type strain (**Figure 4C**). The improvement in plant parameters is correlated with the amount of oxalic acid secreted by *Pf* int1 and *Pf* int2.

## Discussion

Growth and yields of crops in alfisols are low due to acidic pH, aluminum toxicity and limited amount of available P, with high P refixation capacity of the soil [46]. P is strongly complexed with Fe and Al in alfisols, which are difficult to solubilize by mineral acids. Several lines of evidences have shown that certain low molecular weight organic acids can release P from Fe-P and Al-P complexes. Among several organic acids tested, oxalic and citric acid solubilized RP in alfisol [5]. Only few bacteria are known to naturally secrete oxalate but do so in very low amounts as compared to several fungi which secrete oxalate in molar amounts [21]. Thus, the present work was aimed at genetic modification of *P. fluorescens* so as to enable it to secrete high amounts of oxalic acid and render it proficient at solubilizing P from RP amended alfisol. This bacterium was chosen since fluorescent pseudomonads are recognized as plant growth promoting bacteria with efficient root colonizing ability [35], [36]. The strategy used was to express the key enzyme oxaloacetate acetyl hydrolase (OAH) for oxalate synthesis from a fungal system. Presence of this enzyme for oxalate biosynthesis has not been reported so far from bacteria. In order to enable the OAH transformants to secrete the oxalate in the extracellular milieu, an oxalate transporter from another fungal system was deployed.

**Figure 5:**
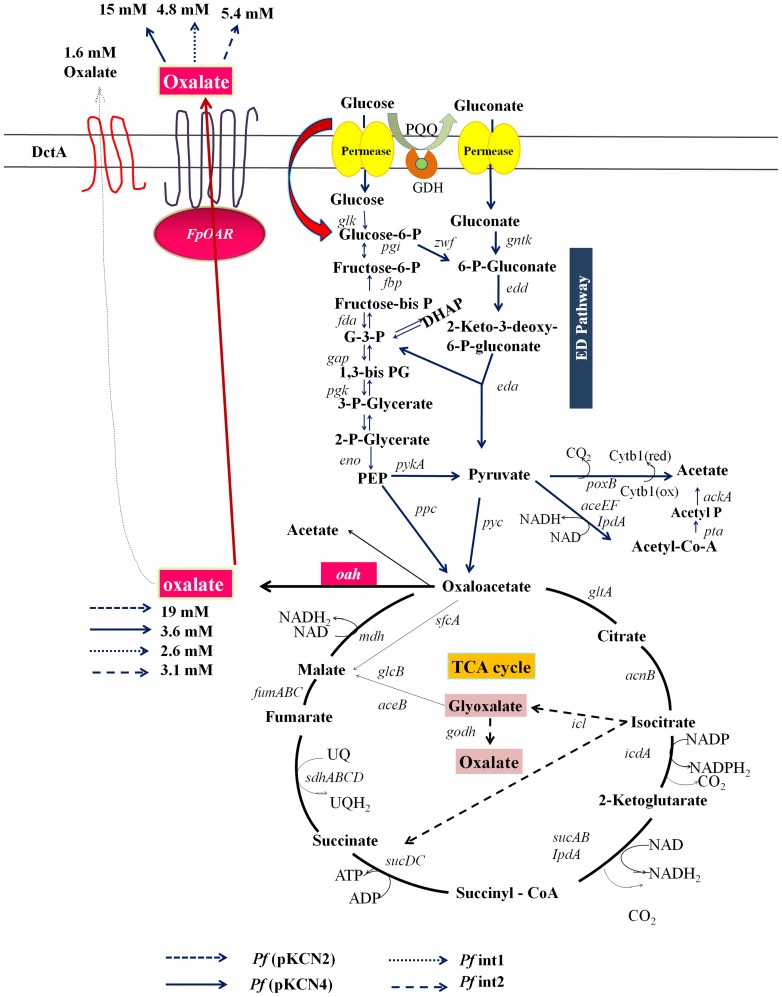
Schematic representation of the genetic modifications of *Pf*13525 and their effect on oxalic acid accumulation and secretion.

*P. fluorescens* ATCC 13525 transformant harboring *oah* gene alone (without the heterologous transporter system) secreted low amount of oxalic acid (**Figure 5**). The ability to secrete the organic acid in the cell free supernatant may be attributed to the resident dicarboxylate transporters (DctA and DctB) [47]. Secretion of oxalic acid by *P. fluorescens* ATCC 13525 has been shown in response to Al toxicity in presence of external citrate [48], suggesting that the resident dicarboxylate transporters are functional. *Pf* (pCNK4) transformant harboring artificial oxalate operon containing *oah* and the fungal transporter *FpOAR* showed enhanced oxalate secretion (**Figure 5**) indicating more efficient transport. The transformant harboring artificial oxalate operon also exhibited MPS phenotype in minimal medium containing RP as the P source and in alfisol. Attaining MPS phenotype in alfisol by oxalate secretion is supported by the fact that 5–10 mM oxalate solubilizes significant amount of RP in alfisols [5]. The oxalic acid secreted by the transformant is also expected to be effective in releasing P from alkaline vertisols which contain high amount of Ca-P [41].

Integration of genes in to the genome is a preferred method for genetic manipulation of bacteria for environmental applications as compared to plasmid transformation, due to increased stability of the integrant, absence of antibiotic resistance genes, lack of horizontal transfer and reduced metabolic load [49]. However, genomic integrants have the limitation of single copy expression leading to weak phenotype. This was reflected in the decrease in levels of oxalate accumulation and secretion in the genomic integrants of artificial oxalate operon as compared to the plasmid transformants (**Figure 5**). However, the amount of oxalate secreted in the integrants was sufficient to solubilize RP in buffered medium as well as in alfisols.

Hydroponic and alfisol soil experiments of *V. radiata* inoculated with genomic integrants demonstrated enhanced root colonization and plant growth as compared to the vector control suggesting that genetic manipulation supported colonization and survival in the rhizosphere. On the other hand, *E. asburiae* PSI3, which is efficient in releasing P from alkaline vertisols mediated by secretion of high levels of gluconic acid, did not improve the growth of *V. radiata* in alfisol [5]. Significant enhancement in plant growth parameters and P content in *Pf* int2 inoculations as compared to *Pf* int1 could be attributed to the presence of VHb which is known to improve the metabolism of bacteria under microaerobic conditions. Similarly, presence of VHb in *Rhizobium etli* increased nitrogenase activity and N content in bean plants [31]. This suggests that metabolism of bacteria in the rhizosphere corresponds to that under microoxic conditions. Since oxalate is also implicated in alleviation of Al toxicity to plants [50] and organic acid secreting rhizobacteria are known to promote plant growth by multiple processes [51], it may be hypothesized that the genetically modified strain could be beneficial to plants in diverse soil conditions.

To summarize, in this study we report the genetic manipulation of rhizosphere colonizing bacterium for the secretion of oxalic acid with the aim of imparting it the ability to carry out mineral phosphate solubilization (MPS) from acidic alfisol to enhance P availability to plants. *Pf*13525 harboring *oah* gene resulted in intracellular accumulation of high amounts of oxalic acid while incorporation of an artificial oxalate operon, containing additionally an oxalate transporter, lead to the secretion of oxalate in the medium, which in turn resulted in MPS ability in the organism. A genomic integrant of artificial oxalate operon showed improved growth and increased P content of *V. radiata* in alfisol soil. Furthermore, presence of VHb contributed to improved root colonization and better survival of *Pf*13525 integrant in soil, thus, improved plant growth and P content. The present work demonstrates the potential of oxalic acid secretion in mineral phosphate solubilization by rhizobacteria.

## Supporting Information

Figure S1 **Schematic representation of arrangement of genes in plasmid constructs used in this study.** (A) Expression plasmid and (B) Integration plasmid constructs. Squares denote the genes, operon and gene clusters cloned in the vector backbone.
(TIF)

Table S1 **Plasmids, bacterial and fungal strains used in this study.** Amp =  ampicillin; Gm =  gentamycin; r =  resistance.
(DOCX)

Table S2 **List of primers used in this study.** Underlined sequences represent restriction enzyme sites used for cloning and sequences in italics indicate universal ribosome binding site (RBS).
(DOCX)
