# Supplementary figures and images for: Pseudomonas fluorescens ATCC 13525 Containing an Artificial Oxalate Operon and Vitreoscilla Hemoglobin Secretes Oxalic Acid and Solubilizes Rock Phosphate in Acidic Alfisols
Source: PLoS One. 2014 Apr 4;9(4):e92400. doi: 10.1371/journal.pone.0092400 (PMC3976251; doi:10.1371/journal.pone.0092400)

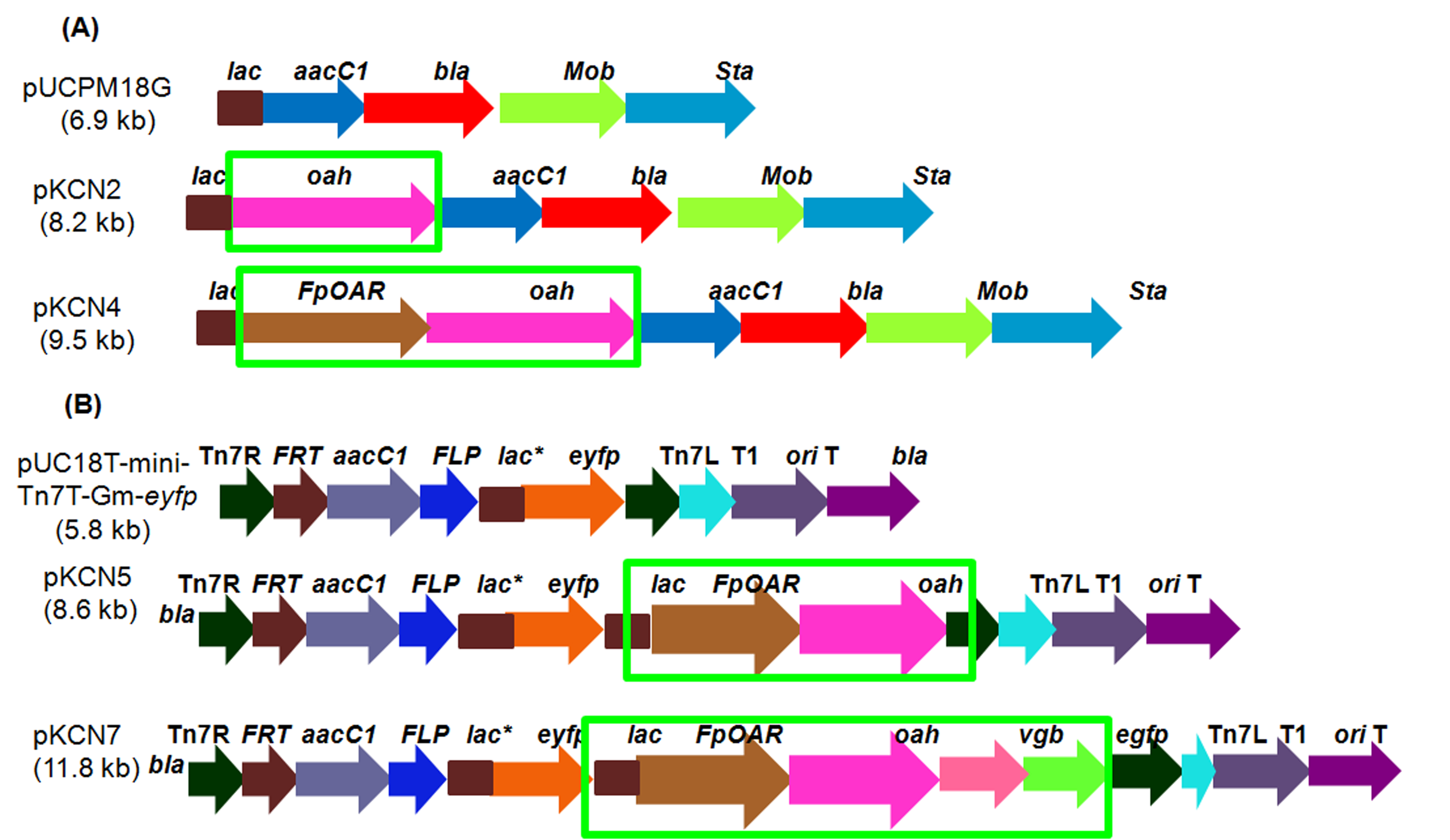

Supplement: Figure S1 — Schematic representation of arrangement of genes in plasmid constructs used in this study. (A) Expression plasmid and (B) Integration plasmid constructs. Squares denote the genes, operon and gene clusters cloned in the vector backbone. (TIF) [file pone.0092400.s001.tif]
