# Supplementary material for: Pseudomonas fluorescens ATCC 13525 Containing an Artificial Oxalate Operon and Vitreoscilla Hemoglobin Secretes Oxalic Acid and Solubilizes Rock Phosphate in Acidic Alfisols
Source: PLoS One. 2014 Apr 4;9(4):e92400. doi: 10.1371/journal.pone.0092400 (PMC3976251; doi:10.1371/journal.pone.0092400)
Supplement: Table S1 — Plasmids, bacterial and fungal strains used in this study. Amp = ampicillin; Gm = gentamycin; r = resistance. (DOCX) [file pone.0092400.s002.docx]

**Supplementary Table 1**

**Plasmids, bacterial and fungal strains used in this study**

| **Strains / Plasmids** | **Relevant Characteristics** | | | | | | **Source/Reference** | |
| --- | --- | --- | --- | --- | --- | --- | --- | --- |
| **Bacterial Strains** | | | | | | | | |
| *P. fluorescens* 13525 | | | | | Wild type | | MTCC, Chandigarh | |
| *Pf* (pUCPM18G) | | | | | *P. fluorescens* 13525 with pUCPM18 plasmid; Amp^r^, Gm^r^ | | This study | |
| *Pf* (pKCN2) | | | | | *P. fluorescens* 13525 with pKCN2 plasmid; Amp^r^, Gm^r^ | | This study | |
| *Pf* (pKCN4) | | | | | *P. fluorescens* 13525 with pKCN4 plasmid; Amp^r^, Gm^r^ | | This study | |
| *Pf* int1 | |  | | Genomic integrant of *P. fluorescens* containing *lac-FpOAR-oah*; Amp^r^ | | This study | | |
| *Pf int* 2 | | | Genomic integrant of *P. fluorescens* containing *lac-FpOAR-oah, vgb, egfp*; Amp^r^ | | | | | This study |
| **Fungal Strains** | | |  | | | | |  |
| 1. *niger* | | | Soil isolate | | | | | This study |
| *Fomitopsis plaustris* | | | Wood rotting fungus | | | | | MTCC, Chandigarh |
| **Plasmids** | | |  | | | | |  |
| pUCPM18Gm | | | pUC18 derived Broad-Host-Range vector; Ap^r^, Gm^r^ | | | | | Lab construct |
| pUC18T-mini-Tn7T-Gm-*eyfp* | | | Mini- Tn7 delivery vector, P *lac*, eyfp; Ap^r^, Gm^r^ | | | | | [41] |
| pKCN2 | | | pUCPM18G with *oah* gene of *A. niger*; Ap^r^, Gm^r^ | | | | | This study |
| pKCN4 | | | pUCPM18G with *oah* gene of *A. niger* and *FpOAR* gene of *F. plaustris*; Ap^r^, Gm^r^ | | | | | This study |
| pKCN5 | | | pUC18T-mini-Tn7T with *oah* gene of *A. niger* and *FpOAR* gene of *F. plaustris*; Ap^r^, Gm^r^ | | | | | This study |
| pKCN7 | | | pUC18T-mini-Tn7T with *oah* gene of *A. niger*, *FpOAR* gene of *F. plaustris*, *vgb* gene of *Vitreoscilla* and *egfp*; Ap^r^, Gm^r^ | | | | | This study |
| pUCVHb-egfp | | | pUC16 with *vgb* gene of *Vitreoscilla*, *egfp*; Amp^r^ | | | | | Lab construct |

Amp= ampicillin; Gm= gentamycin; r= resistance.
