# Supplementary material for: Pseudomonas fluorescens ATCC 13525 Containing an Artificial Oxalate Operon and Vitreoscilla Hemoglobin Secretes Oxalic Acid and Solubilizes Rock Phosphate in Acidic Alfisols
Source: PLoS One. 2014 Apr 4;9(4):e92400. doi: 10.1371/journal.pone.0092400 (PMC3976251; doi:10.1371/journal.pone.0092400)
Supplement: Table S2 — List of primers used in this study. Underlined sequences represent restriction enzyme sites used for cloning and sequences in italics indicate universal ribosome binding site (RBS). (DOCX) [file pone.0092400.s003.docx]

**Supplementary Table 2**

**List of primers used in this study**

| **Primer** | **Sequence (5’ – 3’)** |
| --- | --- |
| *oah* forward primer | CAGGGATCC*CACGGAGGAATCAACTT*ATGAAAGTTGATACCCCCG |
| *oah* reverse primer | GTCGGGCCCCTGCAGTTAGACACCATTAGCAAACC |
| *FpOAR* forward primer | CGAGCTCG*CACGGAGGAATCAACTT*ATGACCGACCTGCATCGA |
| *FpOAR* reverse primer | CGGGATCCCGTCAGAGAAGATCTTCTTG |
| *lac* primer | TTTACACTTTATGCTTCCGGCTCGTATGTTGTGTGGAATTGT GAGCGGATAACAATTTCACACAGGAAACAG CG |

Underline sequences shows restriction enzyme sites used for cloning and sequences in italics are universal ribosome binding sites (RBS).
